# Development of adsorptive membranes by confinement of activated biochar into electrospun nanofibers

**DOI:** 10.3762/bjnano.7.149

**Published:** 2016-11-01

**Authors:** Mehrdad Taheran, Mitra Naghdi, Satinder K Brar, Emile Knystautas, Mausam Verma, Rao Y Surampalli, Jose R Valero

**Affiliations:** 1 INRS-ETE, Université du Québec, 490, Rue de la Couronne, Québec, G1K 9A9, Canada; 2 Département de Physique, de génie physique et d'optique, Université Laval, Québec,G1V 0A6, Canada; 3 CO2 Solutions Inc., 2300, rue Jean-Perrin, Québec, Québec G2C 1T9, Canada; 4 Department of Civil Engineering, University of Nebraska-Lincoln, N104 SEC PO Box 886105, Lincoln, NE 68588-6105, USA

**Keywords:** adsorptive membrane, biochar, chlortetracycline, nanofibers

## Abstract

Adsorptive membranes have many applications in removal of contaminants, such as heavy metals and organic contaminants from water. Recently, increasing concentrations of pharmaceutically active compounds, especially antibiotics, such as chlortetracycline in water and wastewater sources has raised concerns about their potentially adverse impacts on environment and human health. In this study, a series of polyacrylonitrile (PAN)/activated biochar nanofibrous membranes (NFMs) with different loadings of biochar (0–2%, w/w) were fabricated using electrospinning. The morphology and structure of fabricated membranes was investigated by scanning electron microscopy, Fourier transform infrared and thermogravimetric analysis. The results showed that at 1.5% of biochar loading, the surface area reached the maximum value of 12.4 m^2^/g and beyond this loading value, agglomeration of particles inhibited fine interaction with nanofibrous matrix. Also, the adsorption tests using chlortetracycline showed that, under environmentally relevant concentrations, the fabricated adsorptive NFMs had a potential for removal of these types of emerging contaminants from water and wastewaters.

## Introduction

Adsorptive membranes have many applications in clarification, concentration, fractionation and purification processes and offer several advantages over conventional packed bed systems including low backpressure, short residence times and high volumetric throughputs [1]. Adsorptive membranes can be fabricated using membrane precursors with an affinity to target compounds, modification of the membrane surface with functional groups or embedding adsorbents into membrane matrices [2]. There are many reports on functionalization or embedding adsorbents into conventional ultrafiltration membranes for immobilization of different compounds and researchers continuously have tried to improve the performance of adsorptive membranes [3–7]. After demonstration of submicron fibers produced by spinning techniques in 1990s, new horizons emerged for different fields, especially membrane processes [8]. Nanofibrous membranes (NFMs) that are produced by electrospinning can impact the performance of separation technologies because of their high surface to volume ratio, the tunability of pore sizes and the ease of functionalization [9]. Adsorptive NFMs can be used for removal of heavy metals, organic compounds, microorganisms and biomolecules, which makes them ideal candidates for environmental applications. There are many recent reports on the functionalization of NFMs for the removal of compounds of environmental concern. For example, Vanraes et al. used polyamide NFM in combination with electrical discharge to adsorb and degrade atrazine from water [10]. Kampalanonwat and Supaphol and also Neghlani et al. used aminated polyacrylonitrile (PAN) nanofibers to remove heavy metals from water and achieved up to 150 mg/g adsorption capacity for copper [11–12]. Haider and Park fabricated chitosan nanofibers to take advantage of its affinity towards metallic ions, such as copper and lead [13]. In a similar study, Aliabadi et al. used PEO/Chitosan for NFM fabrication to remove nickel, cadmium, lead and copper from aqueous solutions and reported no considerable change in the adsorption capacity after five cycles [14]. Also, there are reports on embedding adsorbent materials into NFMs to enhance the adsorption capability of the composite membranes. For example, Wu et al. [15], Xu et al. [16] and Wang et al. [17] used SiO_2_ particles for the fabrication of composite poly(vinyl alcohol), poly(acrylic acid) and PAN NFMs in order to adsorb metallic ions, malachite green and methylene blue from water. Embedding carbonaceous materials, such as carbon nanotubes and graphene in NFMs has been investigated for different applications, such as glucose sensors, hydrogen storage and enzyme immobilization [18–19]. However, to the best of our knowledge, there is no report on the fabrication of NFM containing carbonaceous adsorbents for the removal of pollutants from aqueous media.

In recent years, emerging contaminants such as pharmaceutically active compounds (PhACs) and endocrine disrupters have been the focus of attention due to their long term effects on human health and environment. Chlortetracycline (CTC), a broad-spectrum antimicrobial agent, is commonly used as veterinary medicine for poultry, swine, and livestock [20]. This compound can enter the environment through the application of animal manure for agriculture, moving to rivers, ground waters and lakes by surface runoff [21]. Presence of CTC and similar compounds in water cycle has raised concerns over potential human health risks. Therefore, removal of PhACs from water sources is necessary. Adsorption of these compounds onto different media, such as carbonaceous materials is one efficient removal method due to feasibility, high efficiency and scalability. Using biochar derived from pinewood for adsorption is of high interest as pine trees account for the majority of forests around the world and more so in Canada. Each year, millions of them are cut and are used for industrial purposes, which produces lots of biomass. Therefore, low cost and high availability make pinewood biomass a promising source for the production of biochar, which is also a value addition strategy for wooden residues [22]. In this study, activated pinewood biochar, with its interesting properties, was incorporated into PAN NFM for the first time to take advantages of both systems. For this purpose, different concentrations of activated biochar (0–2%, w/w) were added into a polymeric solution and the morphological, chemical and thermal properties were characterized. Also, the performance of fabricated membrane for removal of CTC from water was investigated.

## Experimental

### Materials

PAN, with an average weight molecular weight of 1.5 × 10^5^ g/mol, was obtained from Scientific Polymer Product Company (USA) and used without further purification. Biochar was donated by Pyrovac Inc. (Canada) and it was derived from pine white wood (80%) purchased from Belle-Ripe in Princeville and the rest was spruce and fir (20%). This biochar was produced at 525 ± 1 °C under atmospheric pressure for 2 min and used as obtained from the reactor outlet. Sodium hydroxide and hydrogen chloride with 98% purity and *N*,*N*'-dimethylformamide (DMF) and dimethyl sulfoxide (DMSO) with 99.5% purity were supplied by Fisher Scientific (USA). Chlortetracycline (CTC, purity > 97%) was purchased from Toronto Research Chemicals (TRC-Canada). HPLC grade water was prepared in the laboratory using milli-Q/Milli-Ro system (Millipore, USA).

### Activation of biochar

About 20 g NaOH was dissolved in 100 mL of water and 10 g of biochar was added to this solution. The mixture was stirred with a magnetic stirrer (150 rpm) at room temperature for 2 h and it was then dried at 80 ± 1 °C for 24 h. The prepared sample was placed in quartz tube to be heated in a horizontal furnace under nitrogen flow of 200 mL/min. The temperature of the quartz tube was increased to 800 ± 1 °C at 10 °C/min, and held at this temperature for 2 h before cooling down. Later, the product was washed with water, and sodium hydroxide was neutralized with 0.1 M HCl. Finally, for the removal of sodium salt, the product was washed with water and dried at 60 ± 1 °C for 24 h.

### Preparation of PAN-biochar membrane

**Figure 1:**
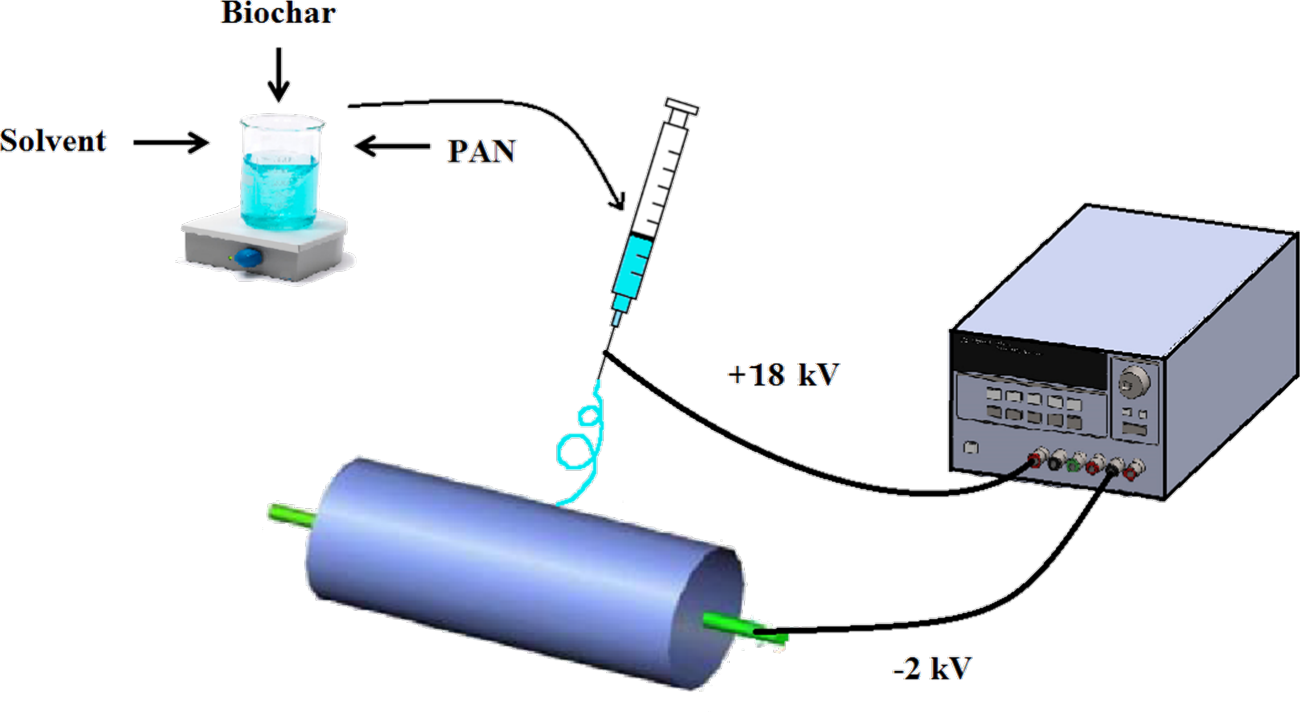
Schematic of the electrospinning system.

A schematic of the electrospinning process for the preparation of NFMs is illustrated in Figure 1. In brief, PAN was dissolved in DMF/DMSO solvent mixture (9:1 v/v) at the concentration of 10 wt % and stirred until a clear solution was obtained. Activated biochar at ratios of 0, 0.5, 1, 1.5 and 2% (w/w) of the polymer was added to the solution and the mixture was stirred for 48 h. Nanofibrous membranes were fabricated via electrospinning under ambient conditions (*T* = 25 °C, RH = 32%) and with a rotary drum collector (length = 25 cm, diameter = 10 cm). The flow rate, electric field strength and collector rotational speed were 1.2 mL/h, 1.1 kV/cm and 400 rpm, respectively. The needle gauge was 22 and the distance of needle tip to the center of collecting drum was 18 cm. The electrospinning continued for 12 h and the deposited mats were soaked in methanol for 60 min to remove residual solvents. The soaked mats were washed with distilled water several times and dried for 10 h at 50 ± 1 °C. To determine the amount of residual solvent, samples for thermogravimetric analysis were not subjected to methanol and heat treatment.

### Characterization of fabricated NFMs

The surface morphology of the fabricated membranes was examined using a JSM-840A (JEOL, Japan) scanning electron microscope (SEM) at an acceleration voltage of 10 kV. For this analysis, small amounts of the samples were coated with a thin layer of gold–palladium alloy using a SPI Module sputter coater. Fourier transform infrared-attenuated total reflectance (FTIR-ATR) spectra were recorded on a Nicolet iS50 spectrometer (Thermo Scientific, USA) at 0.04 cm^−1^ resolution and in the range of 400–4000 cm^−1^. Brunauer–Emmett–Teller (BET) specific surface areas were obtained from the N_2_ adsorption isotherms recorded at 77 K using an Autosorb-1 gas analyzer (Quantachrome Instruments, USA) in the relative pressure range from 0.05 to 1. Thermogravimetric analysis (TGA) and differential scanning calorimetry (DSC) were performed using a STA 449C (Netzsch, Germany) thermogravimetric analyzer. Samples of 10 mg were heated from ambient temperature to 400 ± 1 °C at a constant rate of 10 °C/min under nitrogen with a flow rate of 20 mL/min.

#### Adsorption properties of fabricated NFMs

The capability of fabricated membrane for adsorbing micropollutants was studied on the sample with the highest surface area (NFM-1.5%). Adsorption test was performed on a 15 × 15 cm^2^ stainless steel (SS-316) membrane test module connected to a peristaltic pump. A solution with CTC concentration of 200 ppb in milli-Q water was pumped at a flux of 3 mL/cm^2^·h into the test setup in dead-end configuration. Samples for measuring the CTC concentration were taken at 1 L intervals for 40 L of total passed volume. CTC concentrations were estimated by using laser diode thermal desorption (LDTD) (Phytronix Technologies, Canada) coupled with a LCQ Duo ion trap tandem mass spectrometer (Thermo Finnigan, USA). The daughter ions identified for CTC in LDTD were 464 and 444 Da. The detailed method was explained elsewhere by Pulicharla et al. [23].

## Results and Discussion

### Nanofiber morphology

**Figure 2:**
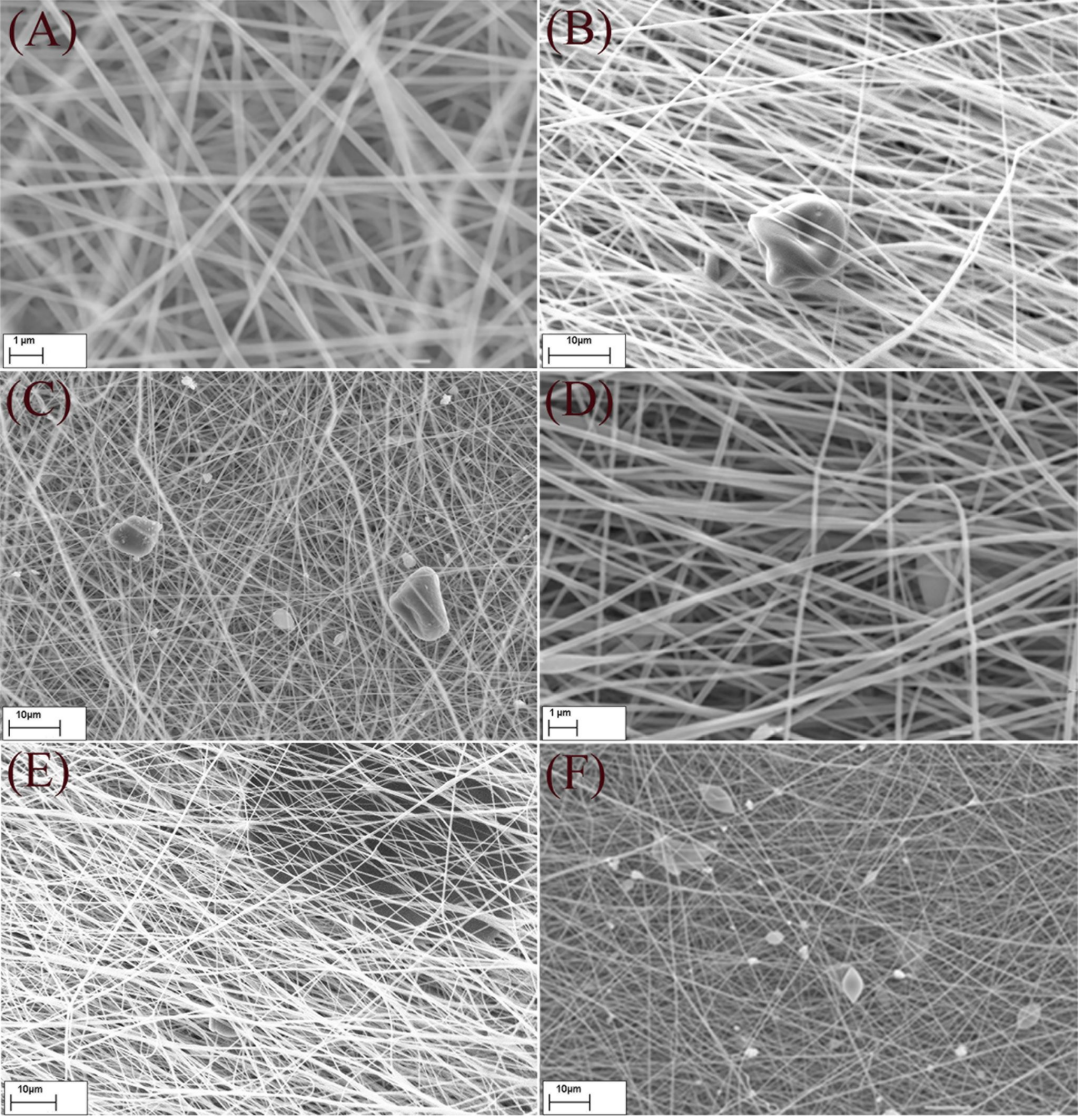
SEM micrographs of NFMs, A) smooth and randomly oriented fibers in NFM-0%, B & C) entrapment of biochar among fibers in NFM-0.5% and NFM-1%, D & E) NFM-1.5% at different magnifications and F) formation of beads in NFM-2%.

The SEM micrographs of fabricated nanofibrous membrane with different contents of activated biochar are illustrated in Figure 2. Generally, the nanofibers were uniform in shape and size and the moderate speed of the rotational drum led to the formation of randomly oriented fibers, which is in favor of membrane fabrications due to required mechanical strength in all directions. Also the entrapment of biochar particles among fibers was perfect since after washing several times with methanol, no leaching was observed visually.

The distribution of the fiber diameter was analyzed using Image-J software with Diameter-J module and the average diameters are listed in Table 1. According to Table 1, the average diameter of fibers was increased from 242 nm for NFM-0% to 316 nm for NFM-2% as the concentration of biochar increased. This can be attributed to the increased viscosity of the solution as a result of adding biochar. Increasing the viscosity of the solution enhances the resistance against being stretched by the charges on the jet and therefore increases the fiber diameter [24]. Generally, an increased viscosity of the solution through addition of more polymer to the solution causes the jet to be more stable and reduce bead formation [25]. However, in this case, viscosity was elevated due to increasing the concentration of biochar particles, which simultaneously disturbed the jet and blocked the needle several times. The particle size of activated biochar was in the range of 5–20 μm. However, there were also few particles with more than tens of micrometer in size. The inner diameter of the employed needle was around 400 μm. Consequently, increasing the content of biochar in polymeric solution will increase the chance of agglomerating big particles in the needle, which may lead to a disturbed jet, clogging of the needle and the formation of biochar aggregates in NFMs. Observing large beads in NFM-2% and also the trend in the BET surface areas of fabricated samples indicated that increasing the fraction of biochar to more than 1.5% disturbed the uniformity of fibers and decreased the surface area. A similar behavior was reported by Ji et al., as they tried to add up to 5% silica to PAN nanofibers and observed beads and particle aggregates for silica loadings above 2% [26].

**Table 1:** Average diameter and BET surface area of fabricated nanofibrous membranes.^a^

sample	activated biochar concentration (%, w/w)	average diameter (nm)	BET surface area (m^2^/g)

NFM-0%^a^	0	242	5.45
NFM-0.5%	0.5	257	5.84
NFM-1%	1	278	9.55
NFM-1.5%	1.5	293	12.52
NFM-2%	2	316	10.87
raw biochar			14.86
activated biochar			853.95
raw PAN			1.14

^a^NFM-0%: nanofibrous membrane with 0% biochar.

### BET surface area

According to Table 1, the enhancement of the specific surface area of electrospun NFMs with increasing biochar content from 0 to 1.5% (w/w) confirmed the entrapment of biochar particles without adverse effect on the fiber structure. However, increasing the biochar content beyond 1.5% caused the formation of large beads that possess a much lower surface to volume ratio compared to a cylindrical geometry and therefore a reduced specific surface area. The statistical analysis confirmed that addition of activated biochar was a significant contributor (p-value = 0.040, F factor = 11.56) to enhancement of specific surface area.

**Figure 3:**
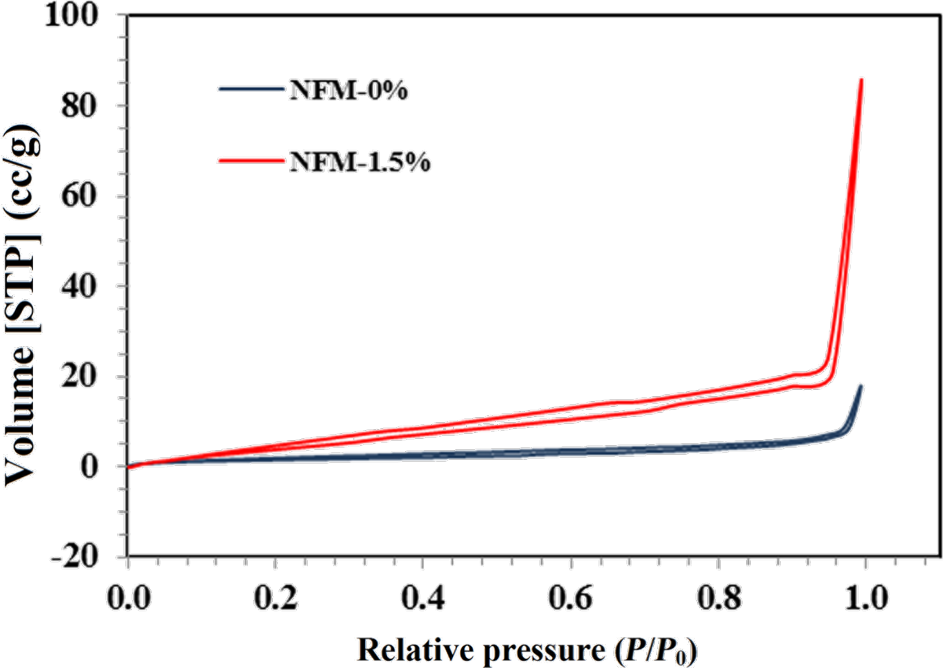
Nitrogen adsorption isotherms at 77 K for NFM-0% and NFM-1.5%.

**Figure 4:**
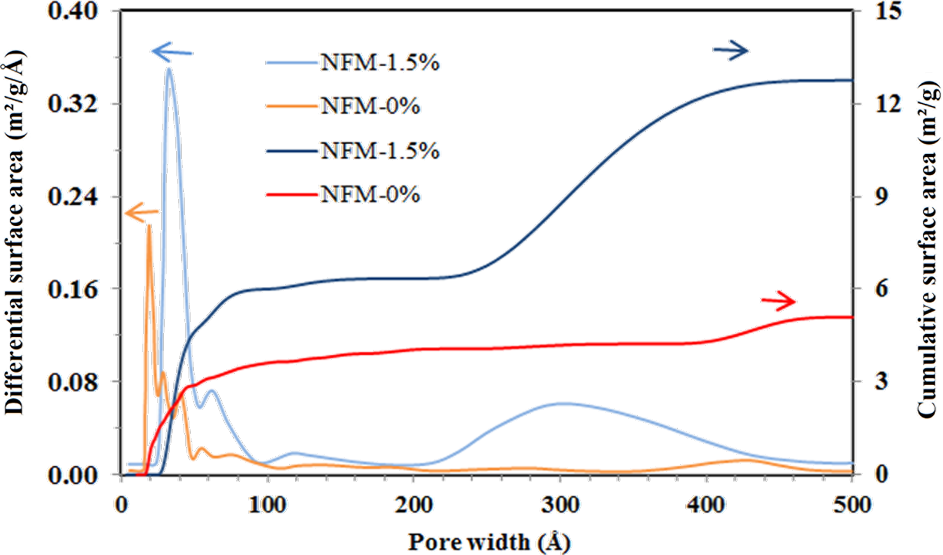
Differential and cumulative surface area versus pore width for NFM-0% and NFM-1.5%.

The nitrogen adsorption isotherms at 77 K against relative pressure and differential and cumulative pore surface area against pore width for NFM-0% and NFM-1.5% are plotted in Figure 3 and Figure 4. The adsorption isotherms indicated that raw NFM-0% had a significantly lower N_2_ adsorption capacity than NFM-1.5%; they had a total pore volume of 0.024 and 0.128 mL/g at 0.99 *P*/*P*_0_, respectively.

According to the differential surface area curves in Figure 4, NFM-0% had pores in two size ranges of 1.5–10 nm and 38–46 nm and the most probable pore size was 2 nm. Also, NFM-1.5% had pores in two size ranges of 2.5–10 nm and 20–50 nm and the most probable pore size was 3.2 nm. The occurrence of pore diameters in the range of 1–10 nm suggested that both samples had pores inside the single fibers. Both samples showed pores larger than 10 nm, which correspond to the interspace between the fibers [27]. Furthermore, the cumulative surface area curves showed that in both samples, around 50% of the surface area corresponded to the pores smaller than 10 nm and 50% corresponded to pores larger than 10 nm.

### FTIR spectroscopy

**Figure 5:**
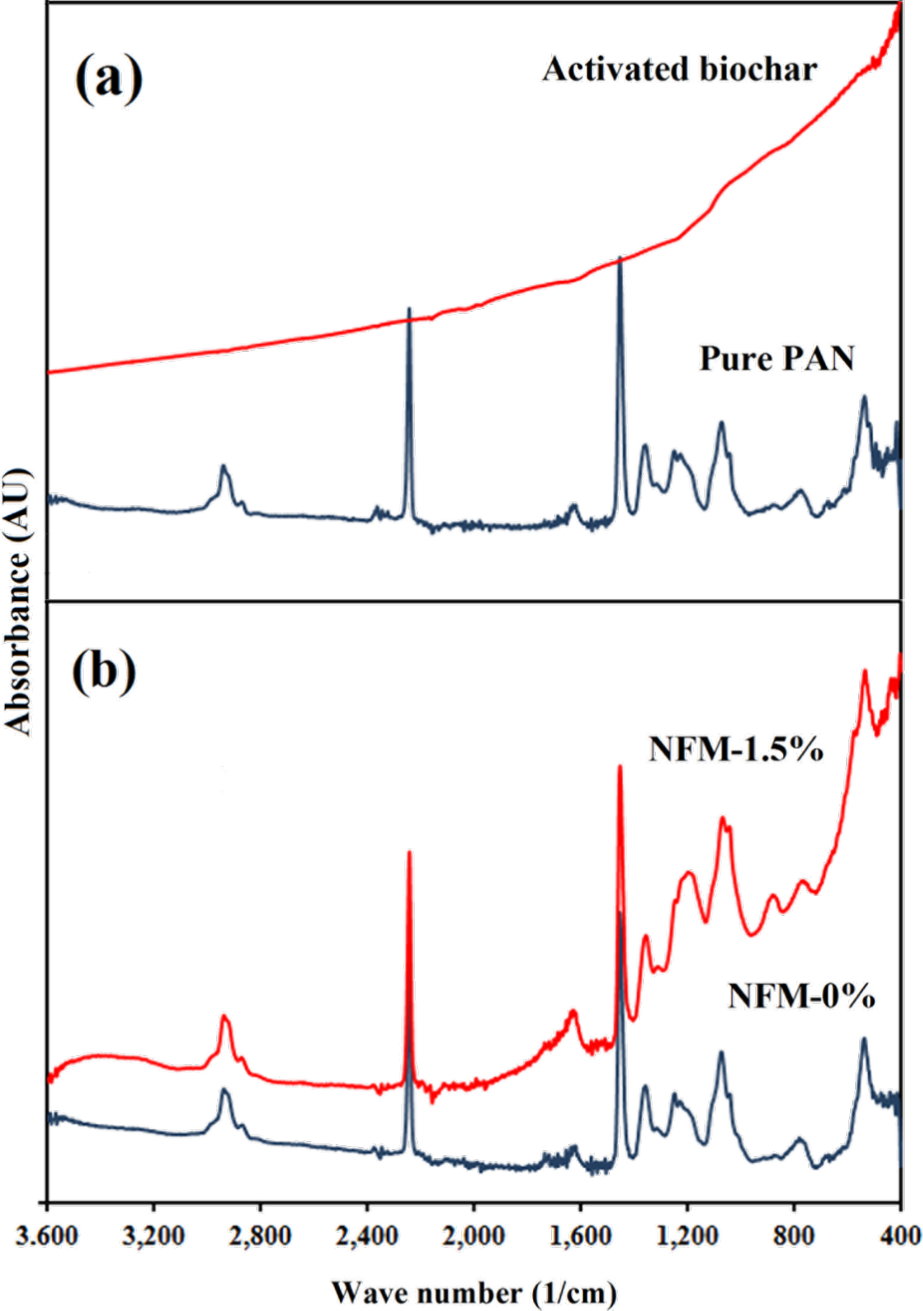
FTIR spectra of (a) pure PAN powder and activated biochar and (b) NFM-0% and NFM-1.5%.

In Figure 5a and Figure 5b, the FTIR spectra of pure PAN powder, activated biochar, NFM-0% and NFM-1.5% are illustrated. Raw biochar (Data are not shown) had two peaks at around 1185 cm^−1^ and 1580 cm^−1^ which correspond to C–H and C=C in aromatic rings [28]. However, activated biochar showed no characteristic peak which indicates that all of the functional groups left the surface during activation.

The PAN molecule consists of nitrile (CN) and methylene (CH_2_) groups in a linear arrangement. The strong IR peak at 2243 cm^−1^ can be assigned to nitrile and the peaks at 1072 cm^−1^, 1453 cm^−1^ and 2940 cm^−1^ are representative for methylene groups [26,29]. Other researchers reported peaks at around 1700 cm^−1^ which corresponded to the carbonyl groups of residual DMF solvent [26]. In this study, due to the methanol washing step, no additional peaks were observed. The characteristic peaks of methylene and nitrile groups were observed in pure PAN powder, NFM-0% and NFM-1.5% at similar wave numbers. However, the pattern for NFM-1.5% was affected by activated biochar so that it looks like a combination of patterns for pure PAN powder and activated biochar.

### Thermal behavior of electrospun PAN nanofibers

**Figure 6:**
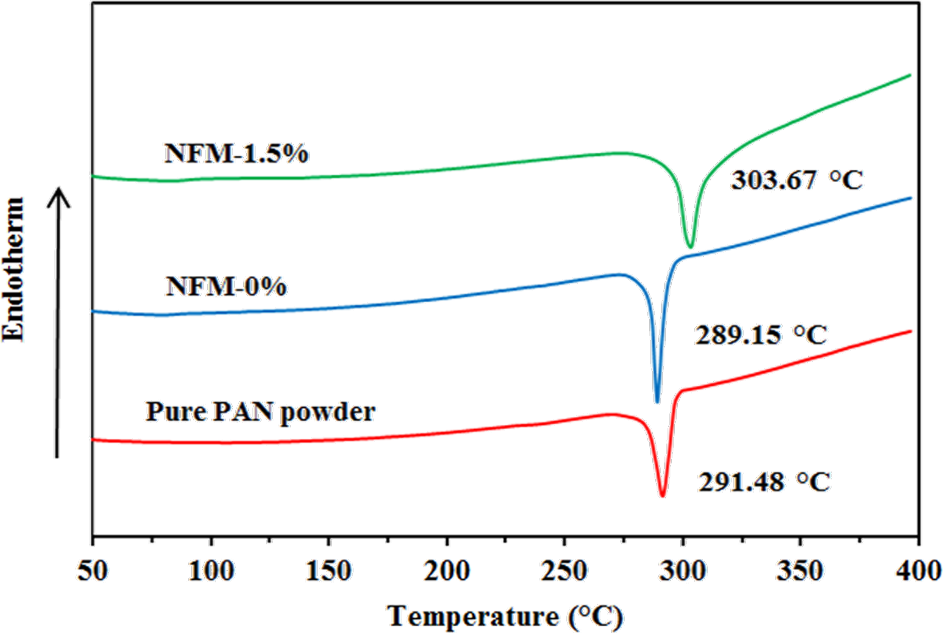
DSC thermograms for pure PAN powder, NFM-0% and NFM-1.5%.

During DSC PAN is heated in the presence of oxygen and begins to degrade near its melting point through an exothermic reaction that can obscure its endothermic melting. Therefore, the melting point cannot be observed for PAN. However, if DSC is conducted in N_2_ atmosphere, the exothermic degradation is observed [30–31]. In Figure 6 the DSC thermograms of pure PAN powder, NFM-0% and NFM-1.5% are illustrated. The sharp peaks located at 291.48 °C, 289.15 °C and 303.67 °C are attributed to the nucleophilic attack at a nitrile and cyclization to an extended conjugated structure [26,30]. The shift of the exothermic peak to lower temperatures from pure PAN powder to PAN nanofiber (NFM-0%) suggests that cyclization is more easily initiated due to molecular rearrangement during electrospinning that resulted in an improved orientation in molecular chains. On the other hand, the shift to higher temperature from NFM-0% to NFM-1.5% confirmed the inhibitory effect of the confined particles [26].

**Figure 7:**
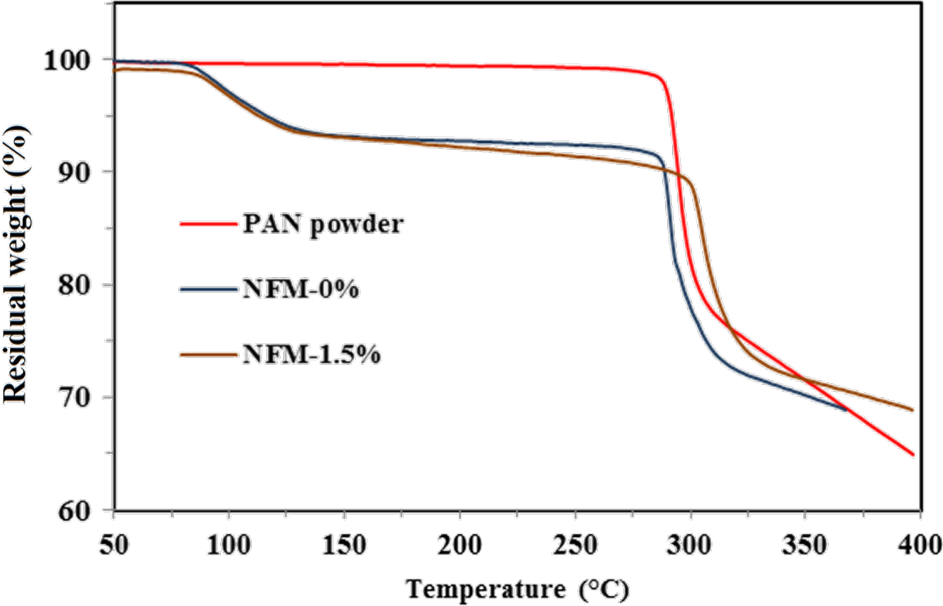
TGA thermograms for pure PAN powder, NFM-0% and NFM-1.5%.

Similarly, Figure 7 illustrates the TGA thermograms of pure PAN powder, NFM-0% and NFM-1.5%. The onset temperature of these samples were 288.9 °C, 286.2 °C and 301.2 °C, respectively, which are in the same order as their exothermic peaks in DSC thermograms. The shifting of onset temperature of electrospun membrane to higher values indicated the strong interfacial interactions between activated biochar and the PAN nanofibers. Also, there was around 8% weight reduction after the temperature exceeded 80 °C for NFM-0% and NFM-1.5% due to evaporation of residual solvents in nanofibers.

### Adsorption properties

**Figure 8:**
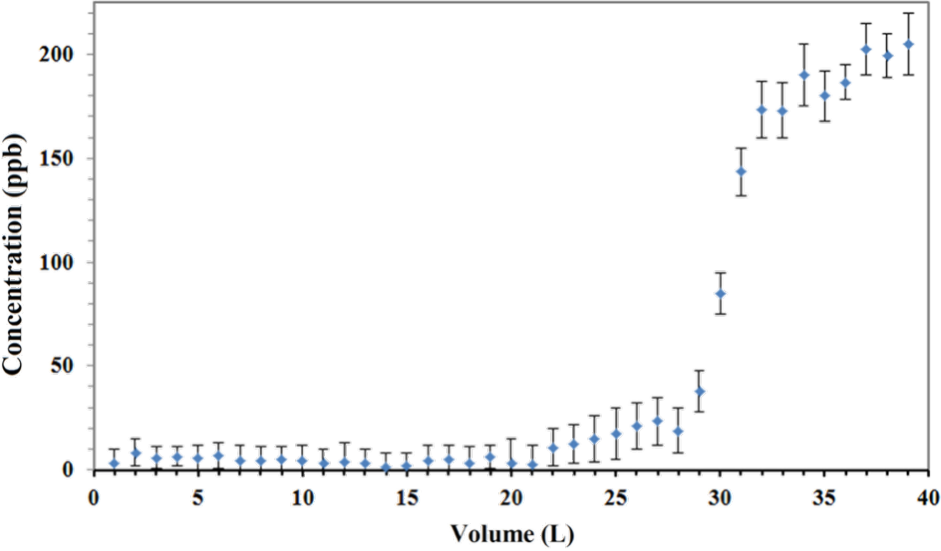
Performance of an adsorptive membrane in the removal of chlortetracycline from aqueous media.

Figure 8 illustrated the performance curve of the fabricated adsorptive membrane with the highest surface area (NFM-1.5%) for removal of CTC from aqueous media. CTC is one of the most hydrophilic veterinary pharmaceutical compounds with log *K*_ow_ = −0.52 [20]. The physicochemical properties of CTC favor its mobility in the environment [32]. In fact, if the adsorptive membrane succeed in removal of CTC, it would be possible to apply for removal of other micropollutants. The CTC concentration in feed stream was set to 200 ppb since the reported values for the influent and effluents of wastewater treatment plants ranged from 1.2 ppb in municipal wastewater to 32 ppm in pharmaceutical wastewater [32–34]. Therefore, the studied concentrations were reasonably in the relevant environmental concentration range. According to the performance curve in Figure 8, more than 95% of the spiked CTC was removed from the first 22 L that passed through the membrane. At first, the entrapped biochar particles are fresh with all their adsorption sites empty and essentially a small part of the target compound can escape. As time passes, some of the adsorption sites are occupied and the concentration in the effluent starts to rise until reaching the same concentration as inlet. From the rising point in Figure 8 it is implied that after passing around 25 L, the membrane should be regenerated. In our previous research, we observed that the adsorption capacity of activated pinewood biochar towards CTC was up to 434 mg/g, which is comparable with graphene oxide and carbon nanotubes [22]. However, in this research, due to the low loading of activated biochar onto membrane, the adsorption capacity was around 6.3 mg/g of membrane. Therefore, further research is still needed to increase the adsorption capacity through increased adsorbent loading or adsorbent specific surface area. Also, working on other applications of these adsorptive membranes, such as immobilization of enzyme would be of interest due to their capability to enhance enzyme loading.

## Conclusion

Adsorptive nanofibrous membranes were fabricated through electrospinning of PAN solutions containing 0–2% activated biochar. SEM micrographs showed that biochar particles were entrapped among nanofibers with diameters in the range of 100–400 nm. The shift of the endothermic DSC peak and the onset temperature of NFM-1.5% compared to NFM-0% and pure PAN indicated interactions between polymer and activated biochar. The results of BET sorption test on fabricated membranes showed that at 1.5% biochar loading, the maximum surface area was obtained. This was due to the aggregation of particles at higher concentrations and also the formation of large beads with reduced surface to volume ratio. Adsorption test in continuous mode indicated that the fabricated membrane can efficiently remove micropollutants, such as CTC from aqueous media. This shows the promising nature of these kinds of systems in removal of emerging contaminants from aqueous environmental streams. However, further research is needed to increase the adsorption capacity of fabricated membranes to compete with commercial adsorbents.
